# Supplementary figures and images for: Upregulation of Mitochondrial Sirt3 and Alleviation of the Inflammatory Phenotype in Macrophages by Estrogen
Source: Cells. 2024 Aug 25;13(17):1420. doi: 10.3390/cells13171420 (PMC11393879; doi:10.3390/cells13171420)

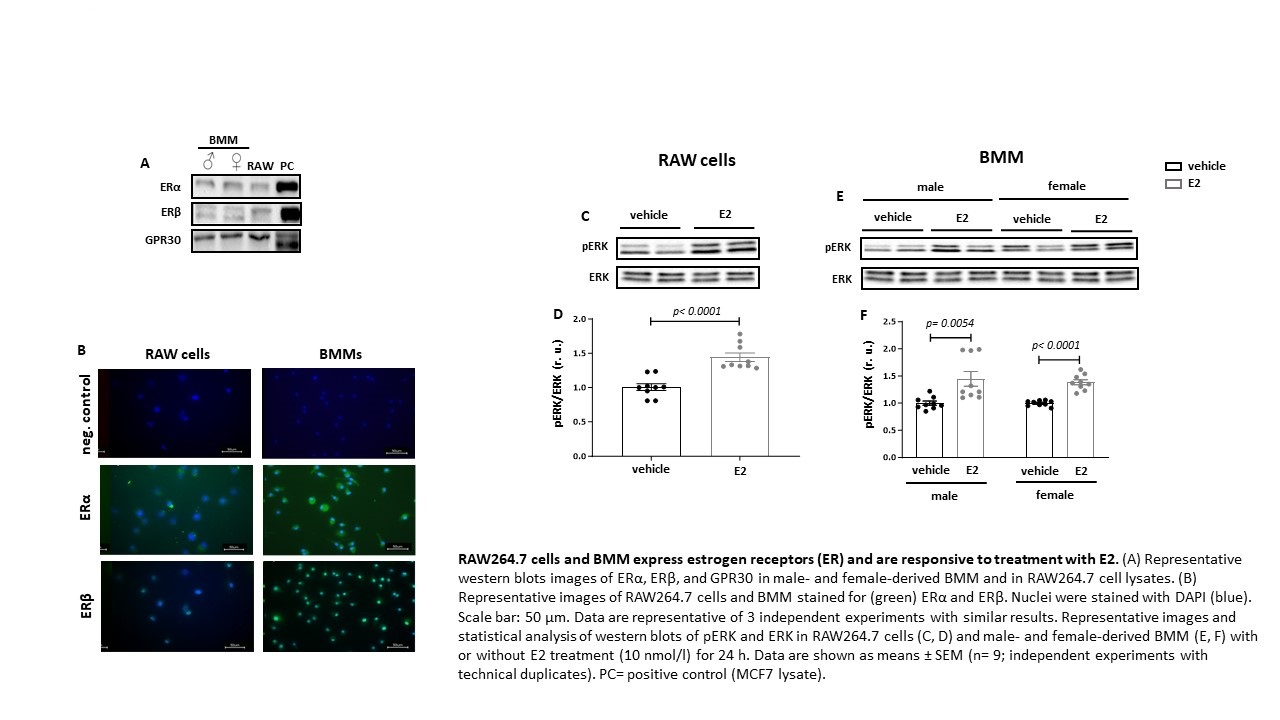

Supplement: Supplementary file 1 [file cells-13-01420-s001.zip › cells-3071064_supplementary/Suppl Figure 2.JPG]

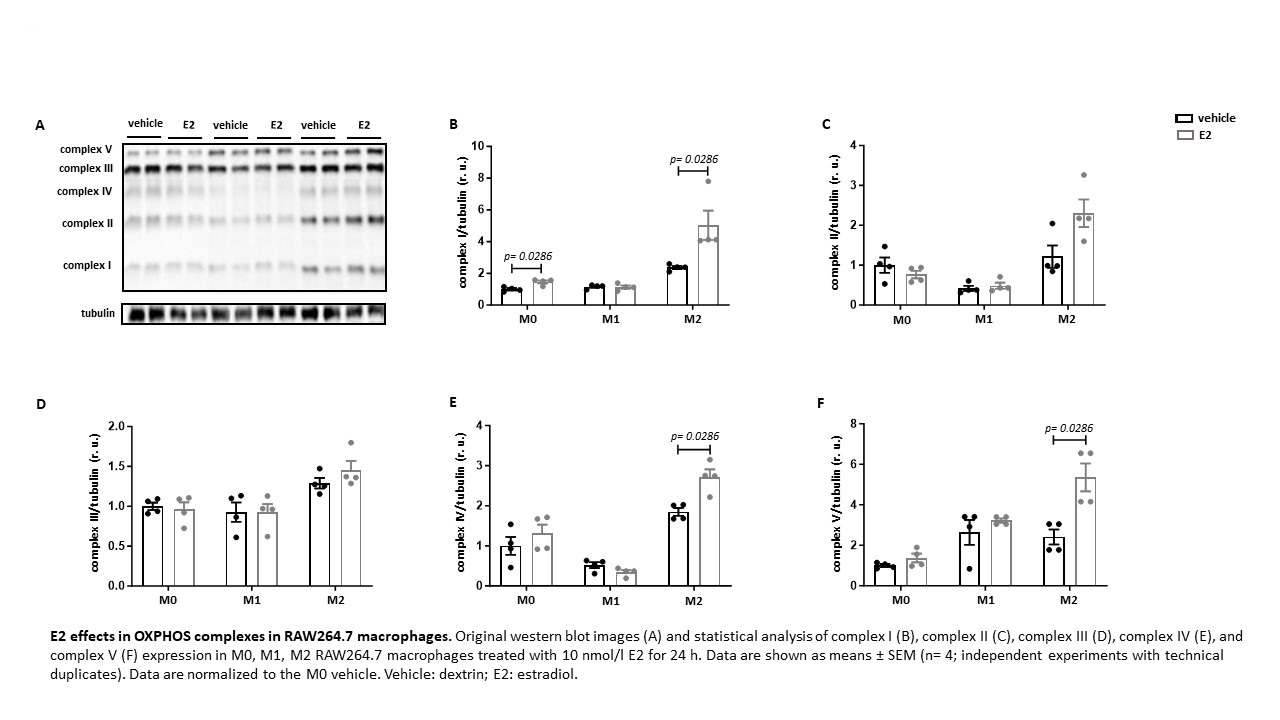

Supplement: Supplementary file 1 [file cells-13-01420-s001.zip › cells-3071064_supplementary/Suppl Figure 4.JPG]

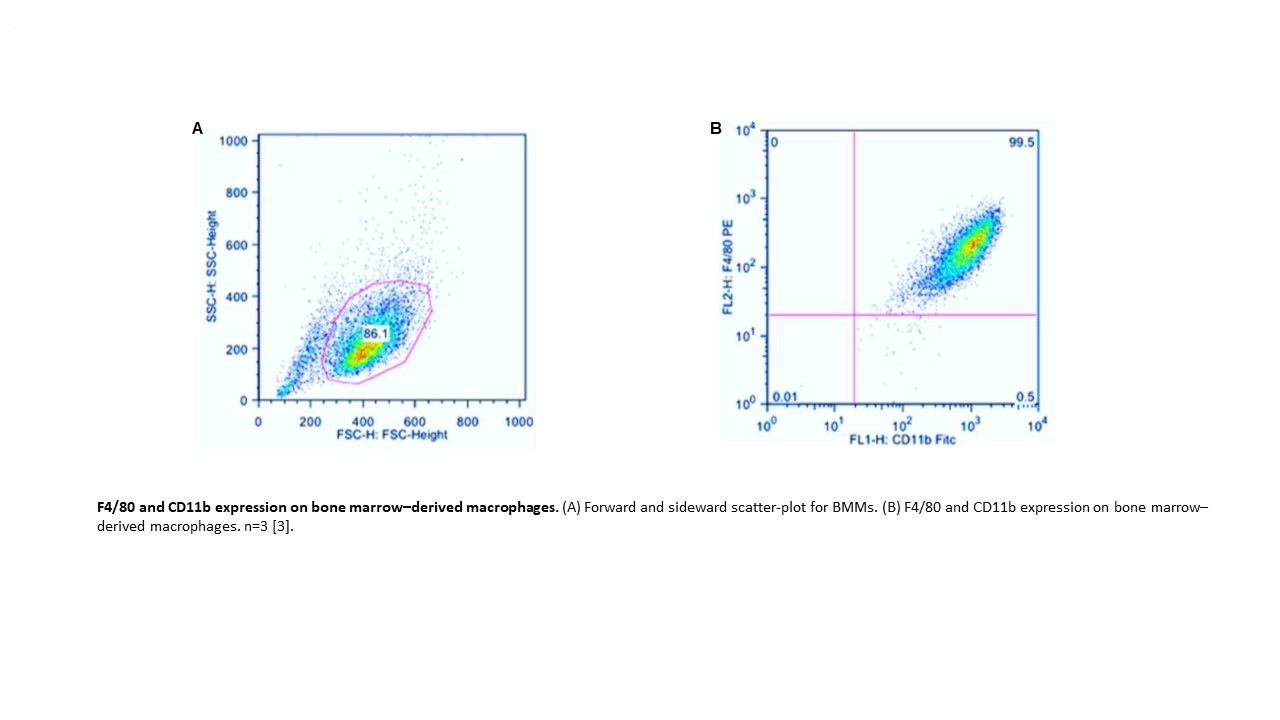

Supplement: Supplementary file 1 [file cells-13-01420-s001.zip › cells-3071064_supplementary/Suppl figure S1.JPG]

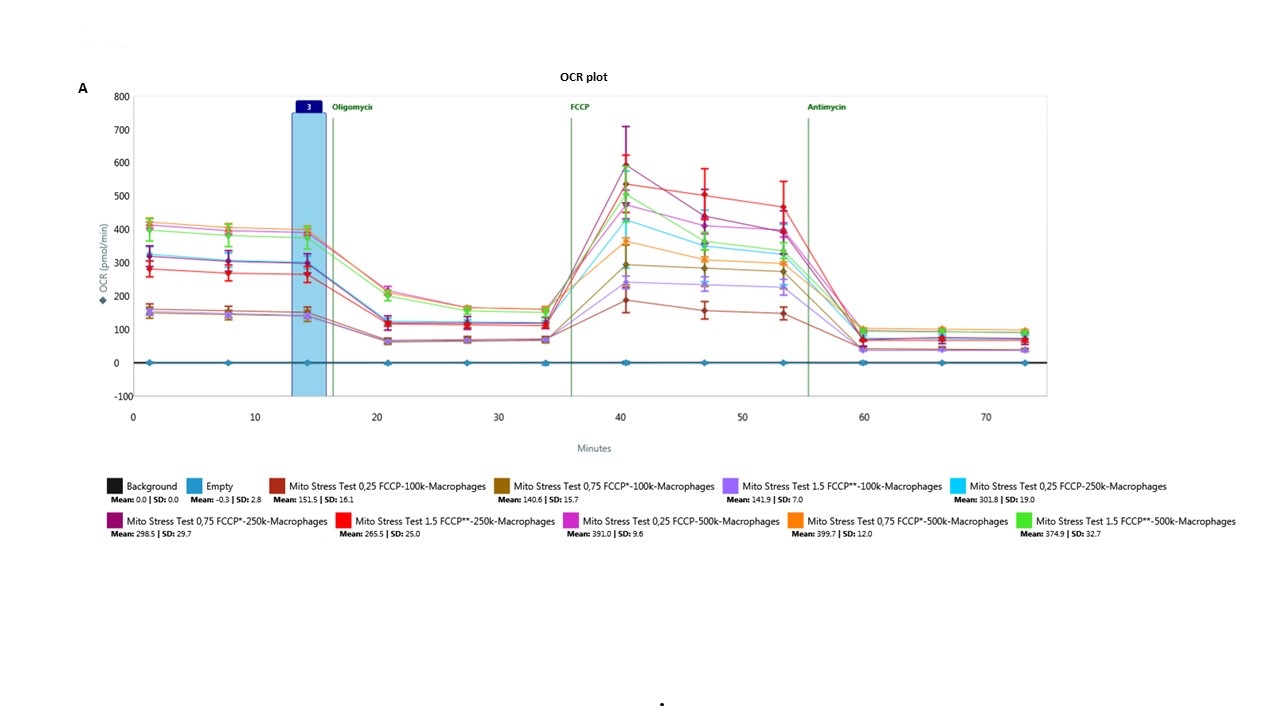

Supplement: Supplementary file 1 [file cells-13-01420-s001.zip › cells-3071064_supplementary/Suppl figure S3.JPG]

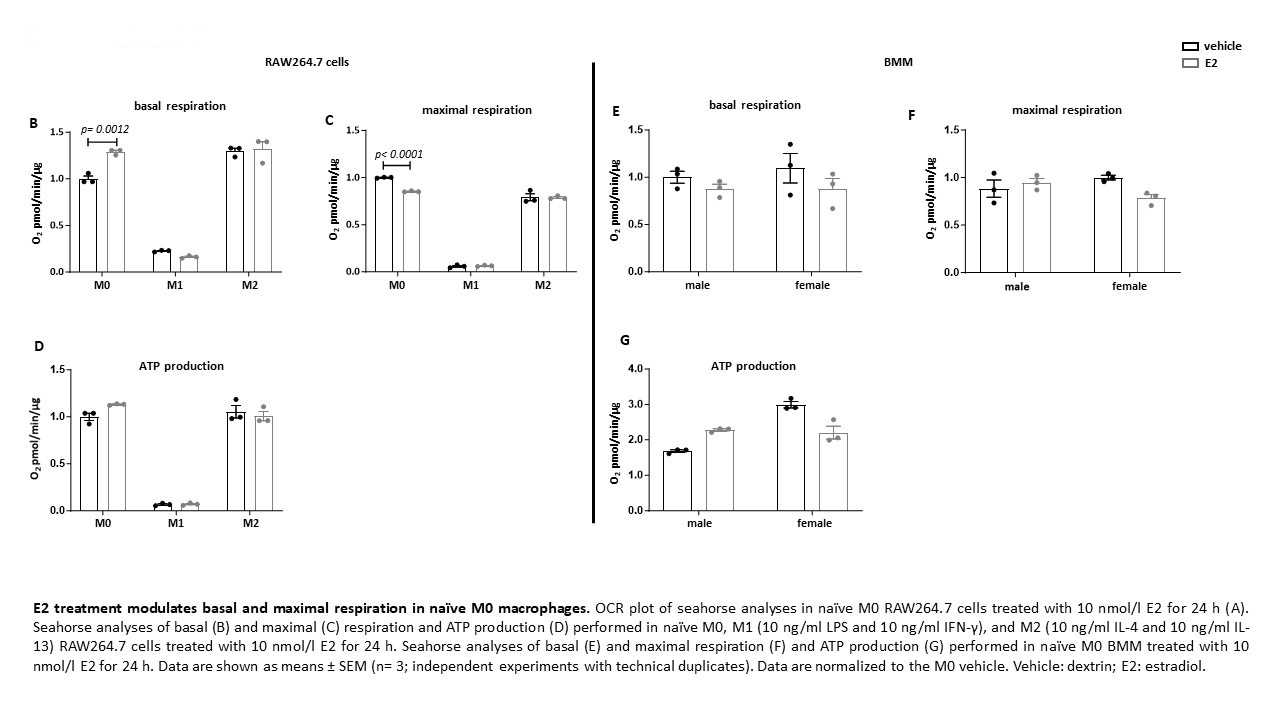

Supplement: Supplementary file 1 [file cells-13-01420-s001.zip › cells-3071064_supplementary/Suppl figure S3extended.JPG]
